# ‘Health is wealth and wealth is health’ – perceptions of health and ill-health among female sex workers in Savannakhet, Laos

**DOI:** 10.3402/gha.v6i0.19080

**Published:** 2013-01-02

**Authors:** Ketkesone Phrasisombath, Sarah Thomsen, Vanphanom Sychareun, Elisabeth Faxelid

**Affiliations:** 1 Department of Postgraduate Studies and Research, University of Health Sciences, Vientiane, Laos; 2 Division of Global Health (IHCAR), Department of Public Health Sciences, Karolinska Institutet, Stockholm, Sweden

**Keywords:** perception, health, ill-health, female sex worker, Laos

## Abstract

**Background:**

Female sex workers (FSWs) are vulnerable to sexually transmitted infections (STIs) and other types of health problems and they also encounter socio-economic difficulties. Efforts to develop effective health intervention programs for FSWs have been hampered by a lack of information on why FSWs do not seek or delay seeking treatment for STIs. To further understand their reasons, our study applied a qualitative approach to explore perceptions of health and ill-health among FSWs in Savannakhet province in Laos.

**Methods:**

Fifteen in-depth interviews were conducted with FSWs in Savannakhet province. Latent content analysis was used for analysis.

**Results:**

Sex workers’ definitions of health and wealth are intertwined. Thus, good health was described as strongly related to wealth, and wealth was needed in order to be healthy. This is explained in two sub-themes: health is necessary for work and income and ill-health creates social and economic vulnerability.

**Conclusions:**

Female sex workers’ beliefs and perceptions about health and ill-health were dominated by their economic need, which in turn was influenced by expectations and demands from their families.

Laos, with a population of 5.6 million, is a low-income country in Southeast Asia. Eighty percent of the population live in rural areas. The literacy rate for the population aged 15 years and above is 73%, with fewer women (63%) compared to men (83%) being literate (1). Seventy-one percent of the population live on less than 2 US$ a day and 23% live on less than 1 US$ a day (2). Due to economic reforms in recent decades, Laos has experienced economic expansion, business transactions, and a blooming of the tourist industry and transportation. However, these changes have also brought domestic and cross-border migration, which has increased social inequalities between people in rural and urban areas. Problems related to poverty have also arisen due to this expansion. Market-oriented growth and urbanization have influenced the lifestyle of ordinary Lao people, and social relations have become more diverse, especially among young people (3).

Despite economic development, Lao traditional culture and norms remain conservative. Sexuality is a sensitive issue and sexual relations outside marriage are strongly discouraged for women. Conversely, pre- and extramarital sex is widely accepted for men, whose sexual needs are perceived as normal and natural (4). A study from Laos found that policy makers and healthcare providers expressed negative attitudes towards young peoples’ sexuality and refused to offer emergency contraceptive pills because of the perception that it would encourage sexual relationships among young people (5). Young women in Laos, especially in rural areas, have limited knowledge about sexuality and reproductive health (4, 6). Gender inequality persists, and when families are stressed financially, girls are often kept out of school. Consequently, women have fewer opportunities for employment and are financially vulnerable, forcing some women to engage in commercial sex as a mean of survival (7).

Commercial sex, although a visible phenomenon in Laos, is illegal. Given the traditional ‘monogamy society’ in Laos, female sex workers (FSWs) are perceived negatively and stigmatized, especially among healthcare providers (8). In Laos, FSWs prefer to be called service women. There are two types of FSWs in Laos: street-based FSWs and venue-based FSWs. Venue-based FSWs are women who are officially employed as hostesses to work in entertainment places (e.g. beer bars ‘drinkshops’, karaoke bars, nightclubs, guesthouses, and restaurants) to provide services to clients in the form of conversation, serving beer and snacks, but also selling sex (9). Venue-based FSWs provide sexual services in guesthouses or hotels or in a room rented by the client, which is usually attached to the entertainment venues. If the woman wants to join the client after the bars close, they can also go together to other settings. The street-based FSWs are women who work outdoors at night on the streets or parks in cities and towns. These women find clients through their own contacts, entertaining them in guesthouses or hotels. Studies from Laos have found that FSWs are vulnerable to sexually transmitted infections (STIs) and other health problems, including adverse consequences of unsafe abortion and violence as well as encountering socio-economic difficulties (7, 10). Furthermore, access to healthcare and information services is limited and influenced by the high cost of treatment and judgmental attitudes of healthcare providers (8, 11).

Laos is a low HIV prevalence country, with 0.2% of adults aged 15–49 estimated to be HIV positive (12). In 2004, an HIV prevalence of 3.9% was detected in FSWs, whereas the prevalence of other STIs such as chlamydia and gonorrhoea in the same group was 33 and 18%, respectively (13). The principle form of STI and HIV transmission in Laos is through sexual contact with an infected partner. The government of Laos has approved a National Strategic and Action Plan on HIV/AIDS/STI for 2011–2015 aimed at expanding national capacity for universal access to treatment, care and support without discrimination (14). The target groups are FSWs, mobile populations and men who have sex with men. Despite these efforts, FSWs in Laos delay seeking care and sometimes do not seek care at all when faced with sexual and reproductive health problems (11). Treatment-seeking behaviour may be affected by several factors, including social norms and health system barriers. It is not known how FSWs in Laos perceive health and illness and how this could be related to treatment-seeking behaviour. Understanding FSWs’ perceptions of health/illness is thus relevant for planning and promoting suitable health interventions for them. In this study, we explore perceptions of health and ill-health among FSWs in Savannakhet province, Laos. This study is part of larger project on perceptions and care-seeking behaviour as well as barriers related to STI services among FSWs in Laos.

## Materials and methods

### Study area

The study was based in Savannakhet province, which has the highest prevalence of STI and HIV infection reported among FSWs in Laos (9). Savannakhet province comprises 15 districts with one provincial and 14 district hospitals. The province has 132,301 households with 826,000 inhabitants, of which women represent 51% (1). The literacy rate of the population aged 15 and above is 68%. About 77% of the population lives in rural areas. The main source of income in the province is from gold mining and cement, sugar production and rubber plants (3). In 2003, care and support for people with HIV/AIDS was initiated in a pilot project providing antiretroviral treatment (ART) in Kaysone Phomvihan, the main district of the province. In addition, a programme called ‘100% condom use program’ was implemented (9). In 2006, a drop-in centre opened in Kaysone Phomvihan. The centre uses a peer education approach and has established services primarily aimed at FSWs. The services include voluntary counselling and testing (VCT) for HIV, and behaviour change and communication (BCC) activities also designated for other groups such as blood donors, migrant workers and hospital patients suspected of having HIV, and those requesting anonymous tests. Condom distribution and STI treatment is also available free of charge to patients. However, if medication is limited or not available, the patients will get a prescription and be requested to buy the recommended medicines in a pharmacy using their own money. In 2007, the VCT program was implemented, and thus far, 21,185 persons have been tested. The cumulative number of HIV positive people in Savannakhet is 1,114, of which 58% are women (15). The majority of those who are HIV positive are FSWs and migrant workers. Kaysone Phomvihan district was chosen as the study site because there are many entertainment places in this district where FSWs work and live. Although there are two types of FSWs in Laos, there are no street-based FSWs in Savannakhet province.

### Study design and research team

A qualitative study using in-depth interviews was performed to gain better understanding about perceptions of health/ill-health among FSWs. A qualitative approach is exploratory in nature and concerned with why the subjects being studied behave as they do, focusing on perceptions, attitudes, beliefs, fears, and experiences (16). The research team comprised of two peer educators who previously used to work as FSWs, one female interviewer who had a background in social sciences, two Lao researchers (KP, VP) with a medical background and experiences in public health research and two Swedish researchers (EF, ST), specialists in international public health research, specifically sexual and reproductive health aspects. The peer educators worked for the drop-in centre and had daily contact with FSWs in the study area distributing condoms and providing health information.

### Procedure and participants

The two peer educators obtained information about the number and type of entertainment places from the drop-in centre. With the support of the peers, the research team member who visited FSWs’ residence and working place contacted FSWs. During a 2-week initiation period, approximately 10 entertainment places in the study area were visited. The Lao research team members took the time to talk and listen to the women describing their health concern, for example, if they had any health problems, their working condition, and their future plan. The women who believed and reported having ill-health for example, complaining about headache, sleeping problem, abdominal pain, vaginal discharge, itching, pain during urinating were recorded for more visits. These first informal discussions with the women created rapport and provided information in order to identify potential women to be interviewed. Our aim was to achieve a sample with maximum variation regarding age, marital status, duration of work as FSW, and type of work place (Table 1). Twenty-one women who reported illnesses including a sexual and reproductive health problem and working as sex workers at the time of the interview were purposively invited for an interview.

**Table 1:** Background of the participants

No	Participants’ working place	Age (year)	Marital status	Duration of working time as FSW
1	Beer bar & nightclub	32	Divorced (2 children)	1 year
2	Beer bar & nightclub	30	Divorced (1 child)	3 years
3	Karaoke bar & guesthouse	25	Single	7 months
4	Beer bar & guesthouse	25	Divorced (1 child)	2 years
5	Beer bar & nightclub	27	Divorced	4 months
6	Beer bar	26	Single	3 years
7	Beer bar & restaurant	24	Divorced (1 child)	2 years
8	Beer bar	21	Single	3 years
9	Beer bar & restaurant	35	Single	10 years
10	Beer bar & guesthouse	18	Single	4 months
11	Beer bar & restaurant	21	Single	1 month
12	Beer bar & nightclub	23	Widow (1 child)	1,5 years
13	Beer bar	25	Divorced (2 children)	1 year
14	Beer bar & restaurant	22	Divorced	1 month
15	Beer bar	25	Married	6 years

Before data collection started, the female interviewer and the peers participated in a 3-day training session on methods of qualitative interviews and how to approach potential interviewees. This training was led by the first author (KP). In order to test the procedure and the interview guide, the interviewer conducted three pilot interviews. The pre-test results were shared with the whole research team and some questions were revised to ensure that the guide was suitable, culturally acceptable, and the words used not too sensitive. Results from these pilots are not included in the analysis. After appointments were made with the potential participants, three could not participate due to time constraints; one moved home without giving any contact information, and two declined because their boyfriend objected. Thus, 15 women were interviewed.

### Data collection

The interviews were conducted during July–August, 2008, in a location convenient for the women where a conversation could be held in privacy (mostly in the women's home or at her work place, i.e. in a living room or behind the bar). The female interviewer explained the purpose of the interview and its procedures. The women were asked to describe what being healthy and having ill-health meant to them, and what they believed signified good/bad health. Each participant was encouraged to discuss issues that were important to them. During each interview, interesting information that emerged was probed and followed up. The interviews were held in Laotian and lasted between 45 and 90 min. The interviews were tape-recorded with the participants’ approval, and notes were also taken.

### Data analysis

The tapes were transcribed into Laotian and then translated into English and shared with the English-speaking research team members in order to validate the analysis. The text was analysed using latent content analysis, which focuses on description and interpretation of underlying meanings of the text (17). This method enables the researcher to compare different concepts in the text toward an accurate understanding of the women's perceptions. The first author initiated the analysis by reading the transcripts of the interviews several times. In order to minimize misinterpretations during reading, the Laotian and English versions were used side-by-side. The meaning units were identified and highlighted. Coding was done initially by the first author and followed by a public health specialist (EF) reading the English transcripts and the codes. The codes that reflected the core meanings of the interview text were identified, and grouped into categories, subthemes and a theme. During analysis, the research team discussed the procedures and the findings until they reached agreement.

### Participant protection

Before the study was implemented, the National Ethics Committee for Health Research, Laos, the Regional Ethics Committee in Stockholm, and the local authority in Savannakhet province approved the verbal consent procedure of the study. Verbal consent was sought from each participant prior to interviewing and they were assured that discussions would be kept confidential. They were also informed that their participation was voluntary and that they could withdraw from the interview at any time without any consequences. No written consent was obtained, reflecting the sensitive nature of sex work and the risk that the informants would fear sanctions as their work is illegal.

## Results

One main theme – ‘health is wealth and wealth is health’ – emerged from the analysis. Health was considered necessary in order to attract clients. On the other hand, money was needed in order to pay for treatment when sick and thus money was a prerequisite for health. The participants’ responses regarding health and ill-health were grouped under two sub-themes: ‘health is necessary for work and income’ and ‘ill-health creates social and economic vulnerability’ (Table 2). Participants perceived health as the ability to work and support their families while ill-health created social and economic vulnerability. The findings are presented below with quotations in order to illustrate these two aspects.

**Table 2:** The analysis process of categories, subthemes and theme

Theme	Health is wealth and wealth is health	
Sub-themes	Health was necessary for work and income	Ill-health creates social and economic vulnerability
Categories	Perceptions of good health	Perceptions of ill-health
	Good health makes one attractive to clients	Ill-health can cause social disapproval
	What contributes to good health

## Health is necessary for work and income

Health was defined as a condition that is needed to be able to work and collect money. Almost all participants reported that good health and physical fitness were necessary to be a sex worker and explained the strong relationship between health and work capacity. Below, we present sex workers’ explanations of how they perceive good health.

### Perceptions of good health

The interviewed sex workers perceived health as the absence of disease especially being free from STIs. One woman explained:

Health is a person who has no disease ‘a condition absent from any sign of a disease’ and not infected with STIs and physically looking well. [Beer bar & nightclub FSW, aged 23]

Some women understood health as a combination of several aspects such as being able to eat, sleep, and sell sex.

The healthy person can sleep, drink alcohol and she can go out ‘having sex’ with many clients. [Beer bar FSW, aged 25]

Most participants reflected that staying healthy was necessary for being able to work. Good health allowed them to be able to work for long hours:

Good health means that you can work and work better than people who are not healthy, you can make a good income. [Nightclub FSW, aged 27]

Another FSW from a beer bar said:

Good health means you don't lose time and do not have to pay for medicine. You can go out and enjoy with your friends. [Beer bar & nightclub FSW, aged 23]

### Good health makes one attractive to clients

Many sex workers explained that good health and physical fitness were necessary to be attractive as a sex worker. As one described:

I think that when I am healthy, clients will see my fresh face, and then they will want me to sit and serve beer for them. Clients like healthy and beautiful girls; they get satisfied and will ask me to go out for sex. [Beer bar FSW, aged 25]

The participants considered that being young and beautiful made them sexually attractive but it was also an essential element to show their health status, which leads to higher income.

Those who are young and beautiful always receive more clients. Clients believe that being young and beautiful is clean, having no disease. Then they will select you. [Karaoke bar & guesthouse FSW, aged 18]

Many women stated that health is important for their business. Those who are healthy usually get higher income than those who are not healthy.

I can see many girls who earn insufficiently because of not healthy, they were sick and had vaginal symptoms. They cannot work, received low income and cannot afford treatment; they had never had a chance. [Nightclub FSW, aged 27]

### What contributes to good health

The participants described several aspects that could support their health. One of the most important aspects was money. Money meant that they could afford healthcare services and pay for other costs when ill. Money was perceived as a vital element, as described in the following quote:

Money can help me being healthy. If I have money I can afford good treatment services when I have health problems. If I don't have money, how can I handle my illness? [Beer bar & restaurant FSW, aged 24]

Some sex workers emphasized that in order to be healthy they should use condoms to prevent diseases when having sex with clients. Also health controls were considered important.

To be healthy I tried to use condoms when having sex with clients. I keep healthy by going for regular health check-ups. [Beer bar & restaurant FSW, aged 35]

The women mentioned that the reason why their health was not good was that they drank alcohol and had sex with many clients every day. Some argued that although money was important, eating adequate and good food was also important for maintaining good health.

What I can do in order to be healthy is eat good food like beef, pork, chicken or fish with some vegetables and also fruit and milk. [Karaoke bar & guesthouse FSW, aged 25]

Some women said that in order to be healthy they needed physical exercise, enough sleep and a clean living environment. However, most women claimed that this was not possible in their current working situation. Some women also mentioned that the best thing for their health would be if they could leave sex work.

### Ill-health creates social and economic vulnerability

The sex workers interviewed emphasized that ill-health affected their ability to work causing financial problems, which in turn limited their ability to support their families. Ill-health also created barriers to socializing when returning home.

### Perceptions of ill-health

Almost all sex workers interviewed defined disease as having an abnormal sign or symptom appearing in their body. They described how a disease could present itself through a feeling or an observable sign as one woman described:

When you have fever or headache it means that you have a disease. [Karaoke bar & guesthouse FSW, aged 25]

Women recognized that having a disease such as an STI has special signs:

I have a *women disease*, ‘STI’. The signs of this disease are discharge, vaginal itching and pain during intercourse. [Beer bar & nightclub FSW, aged 30]

Another sex worker said she knew she had a disease because the doctor had told her when she visited a health clinic:

The doctor told me that I have a disease—‘the vaginal infection’—and needed to take some medicine for a month. [Beer bar & guesthouse FSW, aged 25]

Some participants did not understand what ill-health meant:

Could you explain to me how this is so? I don't know’. [Beer bar & guesthouse FSW, aged 18]

However, other women had a clear view about ill-health and what ill-health meant to them as illustrated in the quote below:

Ill-health is a disease, ill-health means you suffer from diseases such as headache and vaginal discharge. [Karaoke bar & guesthouse FSW, aged 25]

Some sex workers were concerned about the impact of ill-health on their working capacity:

When you get sick you cannot drink beer and provide good services ‘sex with clients’, you cannot earn but you lose. Instead, you have to visit a health facility and pay for treatment services and medicines and you will lose money. [Beer bar & nightclub FSW, aged 23]

Being sick was perceived as a catastrophe by the participants because the bar owners or pimps told sick women to stop working or move out of the bar to avoid their business getting a bad reputation:

When sick, I could not work and have sex with clients. The bar owner asked me to stop working. I wanted to move to another bar but that was not possible because the bar owners would not accept me, they would not accept a sick woman. [Beer bar & restaurant FSW, aged 24]

### Ill-health can cause social disapproval

Fears of one's profession being disclosed and worries about the impact of their work were common. Participants said that they didn't want their family to know about their work as it brought shame and embarrassment to their parents and their family.

When my parents knew about my job, they were very irritated. I was almost killed and they screamed at me ‘We have a bad daughter’. [Beer bar & restaurant FSW, aged 35]

Most women experienced shame and disgrace when ill. They described how people in the villages disapproved of women returning to the village in poor health and often associated ill-health in women with having a ‘bad’ job. This made the women reluctant to go home when ill since it could result in community insult.

When sick I do not go home, because I am afraid that my parents and people in the village where I live will know what happened to me. [Beer bar & restaurant FSW, aged 24]

Some women explained that people in their village would criticize them if they had poor health. This created barriers to socializing because of the need to avoid public insult or embarrassment. One of the women being interviewed explained:

Being sick, you could not participate in the village's activities nor socialize with your friends when you go home. People will come and ask about you. They will suspect that you have a bad job ‘selling sex’ and infected with AIDS. [Beer bar & nightclub FSW, aged 32]

Most participants revealed that although their income was high it was uncertain, and many needed to reserve a portion of their income to support their families as this could release social negative reaction. Meeting one's family's needs could limit the opportunity for using healthcare services. Thus the traditional demand to take care of one's family created a situation where getting sick meant not fulfilling one's duties in society as reported in the quote below:

Many girls who work in this bar do not have enough money when sick because they contribute their income to support their family. Of course, your parents and people in the village will be proud of the support…If they used all the money for healthcare then the family will be in trouble. [Beer bar FSW, aged 26]

Most participants mentioned about delaying treatment or seeking care because they needed to keep their income to support their family. One woman, aged 23, from a beer bar and nightclub reported:

Although I was sick, I have genital symptoms but I will wait to visit health centre. I want to keep my money to support my parents and my siblings. I don't want to spend the money for my health now, I will wait as the symptoms still allow me to work, there are many people were waiting for my support.

Women commonly spoke about paying less attention to their health, that the diseases were not seriously treated, especially STIs. Although a treatment may have been initiated, being under treatment could interrupt their ability to support their family. The health concern in illness of a woman gives the following account:

…according to treatment advised, I use suppository treatment for my abdominal pain and discharge. I still go out for sexual intercourse with clients. What I did is that, when arrived at a guest house, in the toilet, the suppository took out from my vagina before having sex with client…to dilute in vagina it needed at least two days. [Nightclub and guest house girl, aged 25]

## Discussion

According to the sex workers’ accounts, health was clearly associated with the ability to work and earn an income and that they interpreted health mainly in economic terms. The women perceived health as being physically fit, beautiful, and without unpleasant physical symptoms. They also reflected that health was a combination of several aspects such as being able to eat, sleep, and work, for example, selling sex. The perceptions of health as expressed by the FSW in our study obviously differ from the definition of health used by the medical profession (18). Our findings indicate how the women's experiences and concerns about health and ill-health were related to their work. Similar findings have been found from other setting where FSWs interpreted their health in relation to bodily qualities by saying, ‘I am young. My milk is standing. My body is good. No disease’ (19).

There were different perceptions of health described by participants in the present study. Health was important for financial purposes but health was also viewed as a key element in sex work because health was a ‘client demand’ and something that made the sex worker attractive to clients. Our findings are consistent with an ethnographic study in Madagascar (20), where FSWs’ perceptions were influenced by personal identity, socio-economic status and the environmental structure in which they lived. Furthermore, Helman 2007 describes that neither health nor illness are static constructs, but that both are influenced by a particular economic and social situation and also human characteristics, experiences and beliefs (21).

Participants in our study experienced both direct and indirect negative impacts of ill-health. The direct impact reported was economic loss. This is because sex workers can neither take care of the bar duties or have sex with clients when ill, which in turn limits their ability to support their families. The indirect impacts of ill-health were related to social relationships and emotional stress. Sex workers had noticed that people in their society associated ill-health in women who returned home with improper behaviour, for example, selling sex. This created barriers to socializing because of the need to avoid public insult or embarrassment. Sex workers’ descriptions of ill-health indicate the importance of understanding cultural norms and practices relating to ill-health in our study context.

The women not only included their own experiences and perceptions of ill-health, but also the meaning that others around them associated with ill-health. Ill-health often shares the psychological, moral and social dimensions associated with other forms of adversity within a particular society. In a study on tuberculosis in Bangladesh and Vietnam, patients reported feeling ashamed or embarrassed because of the disease. The clinical perspective was less reported, but their feeling of being socially ostracized was a major concern (22, 23). Our findings are important since they suggest that ill-health is a wider, more diffuse concept than just disease. This should be taken into account in understanding how FSWs in Laos interpret their ill-health and how this interpretation might influence their care seeking.

In contexts where there is unequal distribution of wealth and resources, obligations to support one's family are of utmost importance for the survival of the family. The results from a study in Vietnam suggested that FSWs engaged in transactional sex because of the need to support sibling's school fees, paying for a new house, and paying the family's debt (24). A previous study conducted in Pakistan indicated that poverty, financial burdens, and a desire to survive forced women into prostitution (25). Being able to support the family made participants proud and gave them social value. However, this obligation might lead to high-risk sexual behaviour. For instance, when FSWs urgently need money to pay the family's debts or to pay for siblings’ school fees they may force themselves to work harder and also engage in unprotected sex (7). Another study found that when clients refused to use a condom, FSWs charged more money and went ahead having sex without a condom (26).

Many participants indicated that they do not have enough money when sick. They sometimes delayed treatment for STIs in order to reserve a portion of their income for their family. This finding is consistent with results from a previous study conducted by the authors, which showed that 20% of FSWs delayed treatment due to lack of money. The findings also showed FSW's reason in relation to their health, coping with ill-health and experiences of seeking care and treatment—aspects that are closely connected to the women's perception of health/ill-health. Examples for not seeking help included: ‘don't care about the symptoms of STI/not important’, and ‘don't want to visit the health centre because afraid of the result’. Furthermore, reasoning behind not seeking care were, for example, ‘don't know where to go’, ‘judgmental attitude of health care provider’, and ‘inconvenient location’ (11). Poverty limits educational attainment and as a consequence also limits access to the labour market. Women from families with low social and economic status are particularly affected (27). Being a woman in an illegal job means facing enormous obstacles, including high-risk situations and becoming ill, which could result in termination of their work by their employer. Ironically, even though the participants knew that their work was morally wrong and disgraceful to them and their families, they needed to keep the job not only for their own survival but also for the survival of their family.

## Methodological considerations

Using qualitative, in-depth interviews allowed participants to discuss their perceptions, providing insight into their experiences and concerns. Spending time to visit, talk and listen to the women describe their health proved to be a valuable and successful procedure to create rapport so that participants would openly discuss their perceptions about health. In addition, allowing participants to select the venue for the interviews created an environment where the women could freely discuss personal issues. Discussing sex-related matters is sensitive. Therefore, we involved peer educators who used to work as FSWs and a female interviewer with a background in social science to enhance the validity of the findings. Findings from this study stem from women who presently reported ill-health. Therefore, it is the views of those who had a health problem. Those who had left work because of serious illness or were healthy were not included. Despite this, the study provided valuable information about perceptions of health and ill-health among FSWs in Laos. We believe that what the participants shared is also valid for FSWs in other similar settings.

## Conclusions

Female sex workers’ beliefs and perceptions about health and ill-health were dominated by their economic need, which in turn was influenced by expectations and demands from their families. The obligations to support one's family threaten FSWs’ health and social and financial security. Our findings regarding FSWs’ perceptions about health and ill-health can be used in health promotion programs for FSWs.
